# Opioid use may be associated with postoperative complications in myotonic dystrophy type 1 with high-grade muscular impairment

**DOI:** 10.1038/s41598-020-76217-9

**Published:** 2021-01-11

**Authors:** Chan-Sik Kim, Jin-Mo Park, Donghwi Park, Doo-Hwan Kim, Jin-Sung Park

**Affiliations:** 1 Department of Anesthesiology and Pain Medicine, Asan Medical Center, University of Ulsan College of Medicine, 88 Olympic-ro 43-gil, Songpa-gu, Seoul, 05505 Republic of Korea; 2 Department of Neurology, Dongguk University College of Medicine, Gyeongju, Republic of Korea; 3 Department of Physical Medicine and Rehabilitation, Ulsan University Hospital, University of Ulsan College of Medicine, Ulsan, Republic of Korea; 4 Department of Neurology, School of Medicine, Kyungpook National University, Kyungpook National University Chilgok Hospital, 807 Hoguk-ro, Buk-gu, Daegu, 41404 Republic of Korea

**Keywords:** Medical research, Neurology, Risk factors

## Abstract

Individuals with myotonic dystrophy type 1 (DM1) reportedly have a higher risk of postoperative complications than those without DM1; however, factors related to perioperative complications in DM1 patients remain unclear. We aimed to identify the risk factors that may be associated with postoperative complications in DM1 patients. We reviewed medical records of 256 patients with DM1 from 1998 to 2018, among whom 42 (16.4%) had previously undergone 51 surgeries under general and regional anaesthesia. Among the 42 patients, 11 (21.5%) had 13 postoperative complications including respiratory complications, sustained hypotension, wound infection and dehiscence, artery thrombosis and occlusion, and delayed recovery from anaesthesia. There were significant inter-group differences between the non-complicated and complicated groups considering the following parameters: high-grade (≥ 3) muscular impairment rating scale (MIRS), extubation time, postoperative opioid use, and hospital length of stay. Furthermore, univariate analysis revealed that an MIRS score ≥ 3 (odds ratio [OR] 9.346, confidence interval [CI] 1.761–49.595, p = 0.009) and postoperative opioid use (OR 8.000, CI 1.772–36.127, p = 0.007) were the only statistically significant factors. Therefore, clinicians should be cautious in administering opioids, particularly in patients with a high-grade MIRS score during the perioperative period.

## Introduction

Myotonic dystrophy type 1 (dystrophia myotonica; DM1) is a genetic neuromuscular disorder with an estimated global prevalence of 1:20,000. It is primarily caused by the expansion of cytosine–thymine–guanine (CTG) trinucleotide repeat located in the non-coding region of the dystrophia myotonica protein kinase (*DMPK*) gene^1^. Although, it is clinically characterized by myotonia along with facial and distal dominant weakness, it is now categorized as a multisystemic disease involving cataract, diabetes, and arrhythmia, along with diseases of the central nervous system. Patients with neuromuscular diseases such as DM1 reportedly have a higher risk of postoperative complications than those without, this may be attributed to the presence of underlying muscle weakness, scoliosis, and cardiac abnormalities^2^. Additionally, involvement of multiple organ systems and increased sensitivity to anaesthetic medications further increase high risk of postoperative complications^3^. A recent meta-analysis described a predominantly restrictive ventilator pattern observed in DM1, with a significant emphasis on alveolar hypoventilation and chronic hypercapnia^4^. The advent of novel therapeutic strategies targeting DM1 has highlighted the need for a greater understanding of the respiratory decline while focusing primarily on the use of appropriate anaesthetic agents and respiratory management strategies. However, factors related to perioperative complications in patients with DM1 require further elucidation. Therefore, we aimed to address the possible risk factors that may be associated with postoperative complications in DM1 patients who had undergone surgical interventions under general or regional anaesthesia.

## Results

**Figure 1:**
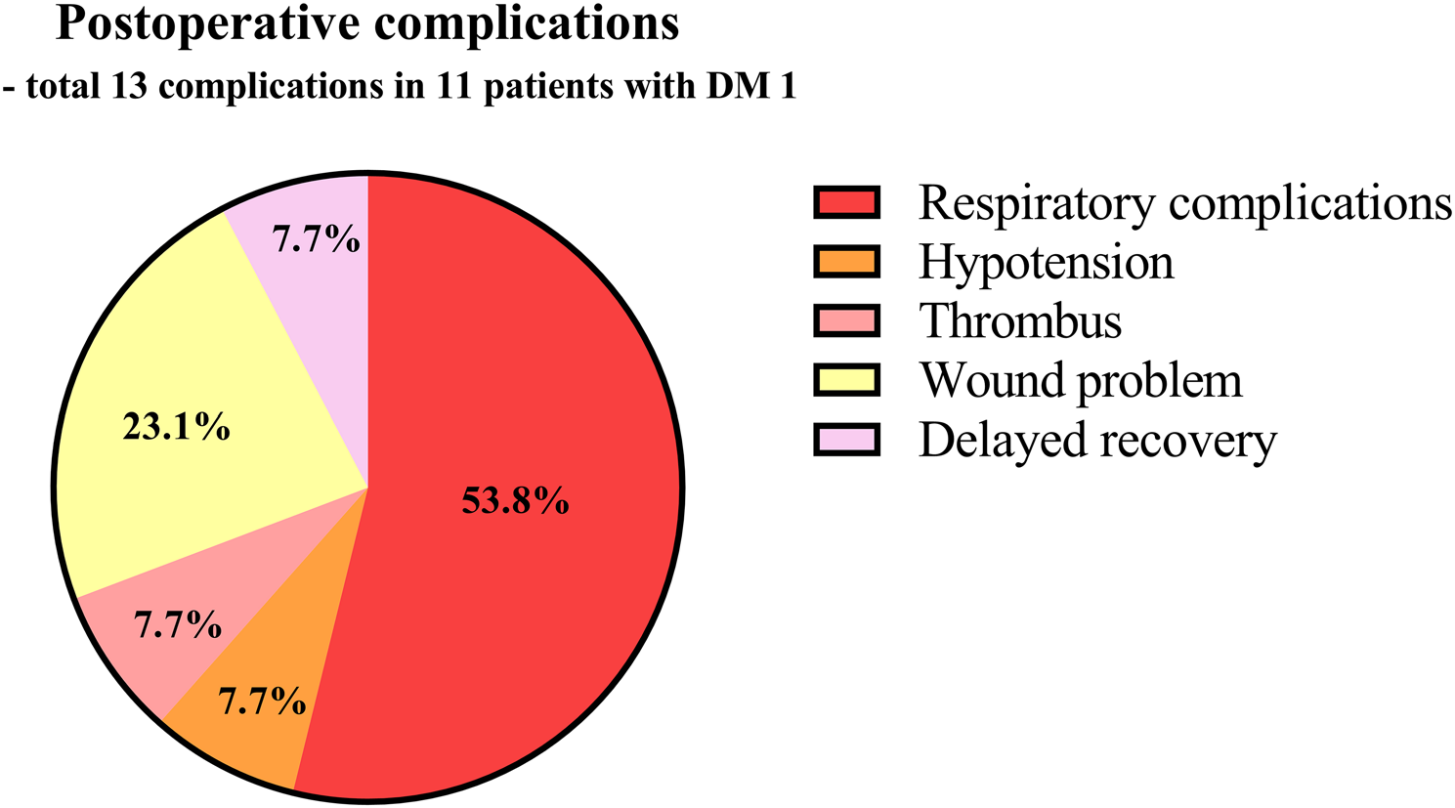
Detailed postoperative complications in patients with myotonic dystrophy type 1.

We enrolled 256 patients with DM1, among whom 42 (16.4%) had previously undergone 51 surgeries under general or regional anaesthesia. The patients were divided into surgical and nonsurgical group as presented in Table 1 and there were no statistically significant inter-differences in the baseline clinical characteristics with regard to their age, body mass index (BMI), CTG repeats, and other comorbid diseases except cataract. However, gender and muscular impairment rating scale (MIRS) score demonstrated statistically significance differences in the baselines for each group. Among the 42 patients, 11 presented with postoperative complications (Fig. 1). Characteristics of the patients with postoperative complications, along with their perioperative parameters, are presented in Supplementary Table S1, S2, and S3.

**Table 1:** Clinical characteristics of the patients with a myotonic dystrophy type 1.

	Non-surgical group (n = 214)	Surgical group (n = 42)	p value
Age (year)	37.0 (28.0–48.0)	36.5 (18.0–43.0)	0.083
Body mass index (kg/m^2^)	21.5 ± 4.3	19.8 ± 3.9	0.016
Gender (male/female)	125 (58.4)/89 (41.6)	13 (31.0)/29 (69.0)	0.002
**MIRS**			0.001
1	2 (1.0)	5 (11.9)	
2	72 (33.6)	16 (38.1)	
3	81 (37.9)	15 (35.7)	
4	51 (23.8)	4 (9.5)	
5	8 (3.7)	2 (4.8)	
**FSRS**			0.361
1	145 (67.8)	31 (73.8)	
2	54 (25.2)	7 (16.7)	
3	11 (5.1)	4 (9.5)	
4	4 (1.9)	0 (0.0)	
CTG repeat size	390 (165–550)	400 (220–540)	0.446
Serum creatinine kinase	293 (166–472)	261 (143–366)	0.153
**Comorbidity**			
Cataract	14 (6.5)	8 (19.0)	0.019
Elevated liver enzymes	29 (13.6)	5 (11.9)	0.969
Autoimmune disease	8 (3.7)	2 (4.8)	> 0.999
Baldness	23 (10.7)	3 (7.1)	0.669
Cardiovascular diseases	5 (2.3)	1 (2.4)	> 0.999
Arrythmia	23 (10.7)	4 (9.5)	> 0.999
Diabetes mellitus	40 (18.7)	3 (7.1)	0.109
Other diseases	11 (5.1)	11 (26.2)	< 0.001

Data are expressed as mean (standard deviation), number (%), or median (interquartile range).

*MIRS* muscular impairment rating scale, *FSRS* functional status rating scale, *CTG* cytosine-thymine-guanine, *Other diseases* Bronchiectasis, myoma, biliary atresia, cryptorchism, hydrocephalus, neurofibroma, and vocal cord palsy.

Opioid administration for postoperative pain control was restricted to almost on the day of surgery (postoperative day 0); additionally, they were treated with nonsteroidal anti-inflammatory drugs or acetaminophen to ameliorate the mild to moderate postoperative pain. However, opioids were administered either in cases of moderate to severe pain associated with orthopaedic or abdominal surgery, or in cases of insufficient analgesia despite the administration of non-opioid analgesics. Eight patients in the complicated group received opioid analgesics on the day of surgery, with the average dosage ranging from 0.3 to 1.9 μg/kg.

We comprehensively compared 42 surgical cases and categorized them into the non-complicated and complicated groups (Table 2). The non-complicated group (n = 31) and complicated group (n = 11) were compared considering their clinical, genetic, laboratory, anaesthesia, and postoperative parameters (Supplementary Table S2). There were no significant differences in their demographic parameters such as age at the time of the procedure operation, BMI, gender, and American Society of Anesthesiologists (ASA) physical status class. The complicated group showed significantly higher occurrences of high MIRS scores (≥ 3) than the non-complicated group, while both groups had similar functional status rating scale (FSRS) scores, CTG repeat lengths, and serum creatinine kinase levels. Both groups showed statistically significant differences in the anaesthesia-related parameters with regard to the extubation time and postoperative opioid use (5.0 min [5.0–13.0] vs. 638.0 min [22.5–1200.0], *p* = 0.003; 7 [22.6] vs. 8 [72.7], *p* = 0.009, respectively). The hospital length of stay (LOS) for the patients in the complicated group was longer than for those in the non-complicated group (5.0 [3.0–8.5] vs. 8.0 [6.5–78.0], *p* = 0.015).

**Table 2:** Comparison of non-complicated group and complicated group during perioperative period.

	Non-complicated group (n = 31)	Complicated group (n = 11)	p value
Age (year)	26.0 (5.5–38.0)	25.0 (15.0–39.5)	0.330
Body mass index (kg/m^2^)	19.5 ± 3.6	18.2 ± 4.4	0.356
Gender (male/female)	8 (25.8)/23 (74.2)	4 (36.4)/7 (63.6)	0.781
**ASA class**			0.214
1	17 (54.8)	4 (36.4)	
2	8 (25.8)	6 (54.5)	
3	6 (19.4)	1 (9.1)	
**MIRS**			0.022
< 3	20 (64.5)	2 (18.2)	
≥ 3	11 (35.5)	9 (81.8)	
**FSRS**			0.560
1	21 (87.5)	8 (72.7)	
2	2 (8.3)	2 (18.2)	
3	1 (4.2)	1 (9.1)	
4	0 (0.0)	0 (0.0)	
CTG repeat size	400 (220–545)	530 (300–550)	0.336
Serum creatinine kinase	264.9 ± 148.9	306.8 ± 157.6	0.434
**Type of anesthesia**			> 0.999
General	28 (90.3)	10 (90.9)	
Regional	3 (9.7)	1 (9.1)	
**Type of surgery**			0.540
Dental	1 (3.2)	0 (0)	
Ears, nose, throat	3 (9.7)	0 (0)	
General	5 (16.1)	4 (36.4)	
Neurology	2 (6.5)	1 (9.1)	
Obstetrics and gynecology	10 (32.3)	5 (45.4)	
Ophthalmology	3 (9.7)	0 (0)	
Orthopedic	5 (16.1)	1 (9.1)	
Urology	2 (6.5)	0 (0.0)	
Muscle relaxants use	25 (80.6)	10 (90.9)	0.754
**Type of anesthetics**			0.994
Inhalation	22 (71.0)	8 (72.7)	
TIVA	6 (19.4)	2 (18.2)	
Intraoperative opioid use	12 (38.7)	5 (45.5)	0.973
Reversal agents use	23 (74.2)	7 (63.6)	0.781
Sugammadex use	3 (9.7)	0 (0.0)	0.697
Airway device	24 (77.4)	10 (90.9)	0.448
ETT	4 (12.9)	0 (0.0)	
**LMA**			
Surgical time (min)	60.0 (40.0–87.5)	85.0 (52.5–155.0)	0.197
Anesthetic time (min)	155.0 (118.5–219.0)	220.0 (164.0–350.0)	0.247
Extubation time (min)	5.0 (5.0–13.0)	638.0 (22.5–1200.0)	0.003
**Postoperative analgesics**			0.009
Others	24 (77.4)	3 (27.3)	
Opioid	7 (22.6)	8 (72.7)	
PACU LOS (day)	50.0 (45.0–58.0)	77.5 (47.5–92.5)	0.227
ICU LOS (day)	2.0 (2.0–2.0)	3.0 (2.5–3.5)	0.081
Hospital LOS (day)	5.0 (3.0–8.5)	8.0 (6.5–78.0)	0.015
Readmission	1 (3.2)	1 (9.1)	> 0.999

Data are expressed as mean (standard deviation), number (%), or median (interquartile range).

*MIRS* muscular impairment rating scale, *FSRS* functional status rating scale, *CTG* cytosine-thymine-guanine, *TIVA* total intravenous anesthesia, *ETT* endotracheal tube, *LMA* laryngeal mask airway, *Others* nonsteroidal anti-inflammatory drugs, acetaminophen, nefopam, and no analgesics, *PACU* Post Anesthetic Care Unit, *LOS* length of stay, *ICU* Intensive Care Unit.

Among 11 patients (complicated group), seven patients had respiratory complications including desaturation [oxygen partial pressure (PaO_2_) < 60 mmHg on room air] and dyspnoea and received prolonged ventilator care for > 1 postoperative day. Three patients presented with wound infection or dehiscence and one with arterial thrombotic occlusion (Supplementary Table S3). One patient who showed delayed recovery, had to recover for three days after the surgery to regain consciousness. One patient had postoperative hypotension. Summarily, there were 13 postoperative complications.

We used univariate regression analysis to identify the factors associated with the postoperative complications in DM1 patients (Table 3). We consequently selected the following parameters for univariate regression analysis considering their clinical importance and statistical significance: age, gender, BMI, MIRS score, hospital LOS, and postoperative use of opioids. MIRS score ≥ 3 and postoperative opioid use were the only statistically significant risk factors with a relatively higher odds ratio (OR), 95% confidence interval (CI), and p value (OR 8.182, 95% CI 1.495–44.772, *p* = 0.015; OR: 9.143, CI 1.899–44.011, *p* = 0.006) (Table 3).

**Table 3:** Univariate regression analysis associated with postoperative complications in patients with a myotonic dystrophy type 1.

Variables	Univariate analysis	
	OR (95% CI)	p value
Age	1.024 (0.980–1.069)	0.286
**Gender**		0.508
Male	1.000	
Female	0.609 (0.140–2.643)	
Body mass index (kg/m^2^)	0.914 (0.757–1.103)	0.348
**MIRS**		0.015
< 3	1.000	
≥ 3	8.182 (1.495–44.772)	
Hospital LOS	1.004 (0.995–1.014)	0.376
**Postoperative analgesics**		
Others	1.000	0.006
Opioid	9.143 (1.899–44.011)	

Data are expressed as odds ratio (95% confidence interval).

*MIRS* muscular impairment rating scale, *LOS* length of stay, *Others* nonsteroidal anti-inflammatory drugs, acetaminophen, nefopam, and no analgesics.

## Discussion

DM1 is clinically characterized by the presence of myotonia, which is an impairment of muscle relaxation following contraction. Electrophysiologically, it can be characterized by the presence of involuntary repetitive action potentials of the muscle membrane that is perpetually in its hyper-excitable state due to a persistent sodium influx or reduced chloride channel conductance^5^. Due to the high prevalence of DM1 in adults, there is an established anaesthesia management guideline based on the recommendations of several experts^6^, which elaborates on the importance of preoperative evaluations of the pulmonary, cardiac, and gastrointestinal symptoms in DM1 patients. It also highlights the need for caution when using anaesthetic and analgesic medications, and the necessity of oxygen saturation and electrocardiography monitoring during the perioperative period. However, these recommendations were based on a limited number of studies, possibly due to the rarity of the disease. Therefore, we aimed to evaluate the possible risk factors influencing DM1 postoperative complications, to consolidate the evidence for the current recommendations suggested by the Myotonic Dystrophy Foundation.

Patients with neuromuscular diseases demonstrate an increased risk of surgical complications when under general anaesthesia due to respiratory muscle weakness, musculoskeletal abnormalities, and cardiac involvement^7–9^. However, only a small number of studies have elaborated on the anaesthesia-related complications in DM1^10,11^. These studies reported that the frequency of postoperative complications in DM1 patients typically ranges from 8.2 to 42.9%^12,13^, which was similar to our study (21.5%) and previous literatures on the subject.

Among the 11 DM1 patients who underwent anaesthesia-related adverse events, 7 (64%) demonstrated respiratory complications and 4 (36.3%) received ventilatory support. A retrospective study that reviewed 219 cases of DM1 reported an 89% prevalence of respiratory complications and 31% required ventilatory support^10^. Another study showed a rate of 10% with regard to postoperative respiratory complications. Discrepancies in the complication rate may be attributable to the severity of the patient’s condition, types of the disease or type of a surgery. It is noteworthy to state that compared to other muscular dystrophies, DM1 appeared to have a significantly high rate of respiratory complications with significantly low cardiac complications. We observed that DM1 patients showed a relatively high complication rate (81.8%) with an MIRS score ≥ 3 and showed an increased odds ratio of 9.346; similarly, Mathieu et al. reported an odds ratio of 14.1^10^. This can be partly explained by the fact that general anaesthesia may have decreased lung compliance and functional residual capacity, leading to alveolar hypoventilation and atelectasis. There is substantial evidence to prove that DM1 patients show abnormalities with regard to the ventilator control mechanisms, and their conditions became more critical when exposed to anaesthetic agents, resulting in a low central respiratory drive^14,15^. Furthermore, this hypothesis can be consolidated by our result in which 9 of 11 patients received abdominal surgery, which is an important risk factor of hypoventilation^16^.

The concerns regarding increase in the risk of defects in the cardiac rhythm conduction and potential progression of known conduction delays in patients with DM1 during the perioperative period are evident^17^. Considering the presence of progressive deterioration of atrioventricular and intraventricular conduction, they can further be exacerbated by anaesthetic drugs, airway manipulation, changes in sympathetic and parasympathetic tone, or hypoxia^2,18^. Although one patient in the complicated group had atrial fibrillation, the risk of cardiac rhythm conduction or aggravated conduction delay remained the same throughout the study. Studies have previously reported that complications associated with perioperative cardiac conduction were absent or were present in only one case^10,11^, which was further validated by our results.

A previous study with 27 juvenile-onset DM1 patients, who had undergone 78 surgeries^11^, Reported that a higher MIRS score, CTG repeat lengths, longer duration of surgery, use of muscle relaxants, and perioperative opioid use were risk factors of perioperative adverse events. However, multivariate regression analysis revealed that a higher MIRS score and use of muscle relaxants were independent risk factors. Interestingly, our result showed that postoperative opioid use and a higher MIRS score were independent risk factors. Although the length of CTG repeats typically correlates with the severity of DM1, we noted no such relationship between the two. The novelty of our study was that we recruited a large number of patients and identified a statistically significant risk factor of postoperative opioid use that influenced the increased possibility of postoperative adverse events in DM1 with an odds ratio of 8.0. Although Sinclair et al. found perioperative opioid use as a risk factor, it could not be considered independent after undergoing regression analysis. The discrepancy may also be explained by differences in mean age of the participants in our study as opposed to that of Sinclair et al. (36.8 years vs 8 years, respectively).

Recent studies have reported the role of central nervous system abnormality in DM1 in the aspects of neurodegeneration^19,20^. Opioid receptors are widely present in brainstem, carotid bodies, the vagus nerve, and airway walls^21^. Opioids upregulate the activity of inhibitory neurons in the brainstem and decrease the rate of respiration and tidal volume^22^; Furthermore, a study has recently reported that administering opioids reduced the ventilator response to hypoxemia and hypercapnia^23^. These mechanisms may have aggravated the overall central nervous system depression to induce greater postoperative complications. However, further investigation is needed to understand the mechanism of high prevalence of respiratory complications in relation to opioids in DM1.

There are a few limitations that need to be addressed. First, the present study was based on a small retrospective analysis, it could limit and compromise its results. However, DM1 is an uncommon genetic neuromuscular disorder, the investigative studies on perioperative outcomes in patients with DM1 are limited. Therefore, we believed that this study further consolidated the known evidence related to the risk factors of perioperative outcomes. Second, this was a retrospective study based on medical and anaesthesia-related records of the patients and therefore selection of analgesics and protocol for analgesic management may be inaccurate, influenced by the preference of individual surgeons and anaesthesiologists. Third, the present study was conducted in only one tertiary hospital and most of the procedures were elective; therefore, the risk of the adverse events might be over- or under-estimated. Lastly, our results do not represent all clinical stages of DM1.

## Conclusions

This is possibly the first study on DM1 patients of Asian ethnicities and our data was in accordance to other Caucasian studies that showed a high prevalence of pulmonary complications after the surgery. Higher MIRS scores positively correlated with a higher prevalence of adverse events. Moreover, postoperative opioid use was also an independent risk factor of accentuated complication rates. Further research on respiratory complications and opioid usage may reduce the rate of onset of surgical complications in DM1.

## Methods

### Patients

The medical records of the patients who visited Asan medical center between January 1998 and June 2018 were reviewed and 256 cases were retrieved. This study was approved by the institutional review board and registered at the University of Ulsan, Seoul, Republic of Korea (2018–0876). We searched information technology of service management of our institution using the terms “myotonic disorders”, “dystrophia myotonica”, and “myotonia congenita”. DM1 diagnosis was based on a genetic study that showed an abnormal increase in the length of untranslated CTG repeat in the *DMPK* gene on19q13. We included DM1 patients who underwent confirmatory genetic examinations; furthermore, DM1 patients who underwent surgery with general or regional anaesthesia were included in the surgical group. We excluded the patients whose diagnosis was not confirmed genetically, along with those who were diagnosed with drug-induced myotonia, pseudomyotonia, and non-drodystrophic myotonia after reviewing their medical data. Patients who had received local anaesthesia during the surgery were also excluded in the surgical group.

### Outcome assessment and factors associated with postoperative complications

The clinical and laboratory parameters of the patients including gender, age at surgery, gender, ASA physical status class, type of surgery, type of anaesthesia, BMI, CTG repeat length, serum creatine kinase, and comorbid diseases including autoimmune disease, cardiovascular disease, arrhythmia, and diabetes, among other parameters were retrieved from their medical records. A functional muscular involvement was evaluated using the MIRS and the FSRS^10^. MIRS is a 5-point scale ranging from 1 to 5, with a higher numerical value reflecting a more severe muscle weakness, with grade 1 = no muscular impairment, grade 2 = minimal signs such as myotonia or facial weakness, grade 3 = distal weakness, grade 4 = mild to moderate proximal weakness, and grade 5 = severe proximal limb weakness. FSRS is a 4-point scale ranging from 1 to 4, in which 1 = mild and 4 = bedridden.

Records related to the type of anaesthesia, hypnotics used for induction, neuromuscular blockers, anaesthetic agents for maintenance, opioid usage, body temperature, reversal agent, airway device, need for extubation, extubation time (minutes) (from the end of surgery to extubation), surgical time (minutes), postoperative analgesics, hospital wards occupied by the patients and duration of hospitalization (post anaesthetic care unit, intensive care unit), hospital LOS (day), re-admission, postoperative complications were retrieved from the medical records. Doses of all opioids administered to patients were converted to intravenous fentanyl equianalgesic doses according to published conversion factors (intravenous fentanyl 100 μg = meperidine 100 mg = tramadol 100 mg)^23^.

^24^Surgery and anaesthesia-associated complications, and any adverse events during the postoperative period (up to seven days after the surgery) were defined as postoperative complications in the study. They may or may not be related to the disease for which the surgery was performed or be the direct results of the surgery^24^. We defined respiratory complications as follows: postoperative PaO_2_ < 60 mmHg on room air, a PaO_2_: fraction of inspired oxygen of ratio < 300 mmHg, arterial oxyhaemoglobin saturation measured with pulse oximetry < 90% and requiring oxygen therapy, or ventilator dependence for > postoperative one day or re-intubation^25–27^. Delayed recovery from anaesthesia was defined as a state of unresponsiveness from which the patient could not be aroused for more than 90 min after being administered with general anaesthesia. We defined sustained hypotension by introducing minor modifications to a definition used in a previous study: systolic blood pressure < 90 mmHg for > 30 min that required the usage of vasopressors^25^.

### Statistical analysis

The data were analysed by using the Statistical Package for the Social Sciences Version 21.0 (SPSS, IBM SPSS Statistics, IBM Corporation, Armonk, NY, USA). Data are expressed as mean (standard deviation), median (interquartile range), number (proportion), or odds ratio (OR) and 95% confidence interval (95% CI). Normal distribution of data was assessed using the Kolmogorov–Smirnov test. Normally distributed continuous demographic data such as body mass index were compared using the Student’s t test; however, non-normally distributed continuous data such as age, CTG repeat size, serum creatinine kinase, and surgical time, among other parameters, were compared using the Mann–Whitney U test. Categorical demographic data were compared using the chi-square test or Fisher’s exact test, as appropriate. By using univariate logistic regression, the factors associated with postoperative complications in patients with DM1 were analysed. A value of *p* < 0.05 was considered statistically significant.

### Ethics

This study was performed according to the Declaration of Helsinki. The current study protocol was approved by the institutional review board of Asan Medical Center, Seoul, Korea (approval number: 2018-0876). Due to the retrospective nature of the study, informed consent was waived.

## Supplementary information

Supplementary Information.

## Data Availability

The datasets generated during and/or analysed during the current study are available from the corresponding author on reasonable request.
