# Supplementary material for: Opioid use may be associated with postoperative complications in myotonic dystrophy type 1 with high-grade muscular impairment
Source: Sci Rep. 2021 Jan 11;11:8. doi: 10.1038/s41598-020-76217-9 (PMC7801477; doi:10.1038/s41598-020-76217-9)
Supplement: Supplementary file 1 — Supplementary Information. [file 41598_2020_76217_MOESM1_ESM.docx]

**Opioid use may be associated with postoperative complications in myotonic dystrophy type 1 with high-grade muscular impairment**

Chan-Sik Kim^1,a^, Jin-Mo Park^2,a^, Donghwi Park^3^, Doo-Hwan Kim^1*^, Jin-Sung Park^4*^

^1^Department of Anesthesiology and Pain medicine, Asan medical center, University of Ulsan college of Medicine, Seoul, Republic of Korea; ^2^Department of Neurology, Dongguk University College of Medicine, Gyeongju, Republic of Korea; ^3^Department of Rehabilitation, Ulsan University hospital, Ulsan, Republic of Korea; ^4^Department of Neurology, School of medicine, Kyungpook National University, Kyungpook National University Chilgok hospital, Daegu, Republic of Korea

^a^These authors contributed equally to this work.

*Co-correspondences: Doo-Hwan Kim, Department of Anesthesiology and Pain Medicine, Asan Medical Center, University of Ulsan College of Medicine, 88 Olympic-ro 43-gil, Songpa-gu, Seoul 05505, Republic of Korea. (Tel: +82-2-3010-1417, Fax: +82-2-3010-6790, e-mail: knaaddict@gmail.com); Jin-Sung Park, Department of Neurology, Kyungpook National University Chilgok Hospital, 807 Hoguk-ro, Buk-gu, Daegu, 41404, Republic of Korea. (Tel: +82-53-200-2753, Fax: +82-53-200-2029, e-mail: [neurojspark@gmail.com](mailto:neurojspark@gmail.com))

**Supplementary Table 1.** Preoperative characteristics of the patients with postoperative complications

|  | Preoperative period | | | | | | | | |  |
| --- | --- | --- | --- | --- | --- | --- | --- | --- | --- | --- |
| No | Age (year) | Gender  (male/female) | BMI  (kg/m) | Muscular Impairment  Rating Scale | Functional Status  Rating Scale | CTG  repeat size | Serum  creatinine kinase | ASA class | Surgery - Type | |
| 1 | 42 | 0 | 25.9 | 2 | 1 | 300 | 543 | 2 | Orthopedic | |
| 2 | 23 | 1 | 15.6 | 3 | 2 | 550 | 167 | 2 | Obstetrics & gynecology | |
| 3 | 25 | 1 | 15.6 | 3 | 2 | 550 | 372 | 2 | Obstetrics & gynecology | |
| 4 | 13 | 0 | 14.9 | 3 | 1 | 1500 | 490 | 1 | General surgery | |
| 5 | 13 | 0 | 15.4 | 3 | 1 | 1500 | 466 | 1 | General surgery | |
| 6 | 41 | 1 | 16.4 | 3 | 1 | 550 | 347 | 1 | Obstetrics & gynecology | |
| 7 | 56 | 0 | 21.3 | 3 | 1 | 220 | 297 | 1 | General surgery | |
| 8 | 36 | 1 | 21.1 | 2 | 1 | 300 | 30 | 3 | General surgery | |
| 9 | 38 | 1 | 21.0 | 5 | 3 | 400 | 143 | 2 | Obstetrics & gynecology | |
| 10 | 0 | 1 | 10.8 | 3 | 1 | 150 | 270 | 2 | Neurosurgery | |
| 11 | 45 | 1 | 22.5 | 3 | 1 | 530 | 250 | 2 | Obstetrics & gynecology | |

BMI, Body mass index; CTG, cytosine-thymine-guanine; ASA, American Society of Anesthesiologists

**Supplementary Table 2.** Intraoperative anesthetic variables of the patients with postoperative complications

|  | Intraoperative period (Anesthesia) | | | | | | | | | | |
| --- | --- | --- | --- | --- | --- | --- | --- | --- | --- | --- | --- |
| No | Type | Induction | NMB | Maintenance | Opioid | Body Temp (˚) | Reversal agent | Airway | Extubation | Extubation  time (min) | Surgical  time (min) |
| 1 | GA | Pentotal sodium | Vec | Enf + N_2_O | None | 36.2 | None | ETT | 1 | 25 | 185 |
| 2 | GA | Pentotal sodium | Esm | Sevo | None | 36.3 | Pyrido | ETT | 0 | 2900 | 50 |
| 3 | EA | - | - | Epidural | Fent | 36.5 | - | - | - | - | 30 |
| 4 | GA | Etomidate | Esm | Sevo | Fent | 36.5 | Pyrido | ETT | 0 | 1235 | 55 |
| 5 | GA | Propofol | Vec | TIVA | Remi | 36.2 | Pyrido | ETT | 0 | 1360 | 125 |
| 6 | GA | Propofol | Esm | TIVA | Remi | 37.0 | Pyrido | ETT | 1 | 20 | 222 |
| 7 | GA | VIMA | Vec | Sevo + N_2_O | None | 36.0 | Pyrido | ETT | 1 | 30 | 85 |
| 8 | GA | Etomidate | Vec | Sevo | None | 37.7 | Pyrido | ETT | 1 | 5 | 250 |
| 9 | GA | Pentotal sodium | Esm | Des + Remi | Remi | 35.2 | None | ETT | 0 | 795 | 90 |
| 10 | GA | Pentotal sodium | Vec | Sevo | None | 37.0 | None | ETT | 0 | 1165 | 40 |
| 11 | GA | Pentotal sodium | Esm | Sevo + N_2_O | None | 36.6 | Pyrido | ETT | 1 | 5 | 80 |

GA, General anesthesia; EA, Epidural anesthesia; VIMA, Volatile Induction and Maintenance Anesthesia; NMB, Neuromuscular blocker; Vec, Vecuronium; Esm, Esmeron; Enf, Enflurane; Sevo, Sevoflurane; Des, Desflurane; TIVA, Total intravenous anesthesia; Remi, Remifentanil; Fent, Fentanyl; Pyrido, Pyridostigmine; ETT, Endotracheal tube.

**Supplementary Table 3.** Postoperative variables of the patients with postoperative complications

|  | Postoperative period | | | | | | |
| --- | --- | --- | --- | --- | --- | --- | --- |
| No | Postoperative opioid use | Discharge | PACU stay (min) | ICU stay (day) | LOS (day) | Re-admission | Postoperative complications |
| 1 | Meperidine | PACU | 30 | - | 17 | None | Wound infection |
| 2 | None | ICU | - | 3 | 9 | None | Delayed recovery |
| 3 | Fentanyl | ICU | - | 2 | 7 | None | Respiratory complications |
| 4 | Meperidine | ICU | - | 14 | 144 | None | Respiratory complications |
| 5 | Meperidine | ICU | - | 4 | 144 | None | Respiratory complications,  Wound dehiscence |
| 6 | None | ICU | - | 3 | 6 | None | Respiratory complications, hypotension |
| 7 | Tramadol | PACU | 65 | - | 8 | None | Respiratory complications |
| 8 | Fentanyl | PACU | 90 | - | 139 | None | Thrombus, arterial occlusion |
| 9 | Meperidine | ICU | - | 3 | 8 | None | Respiratory complications |
| 10 | Fentanyl | ICU | - | 2 | 6 | Yes | Wound infection |
| 11 | Fentanyl | PACU | 95 | - | 6 | None | Respiratory complications |

PACU, Post Anesthetic Care Unit; ICU, Intensive Care Unit; LOS, Length of stay; Respiratory complications, ventilatory care, dyspnea, and desaturation.
